# Impact of tensile strain on low Sn content GeSn lasing

**DOI:** 10.1038/s41598-018-36837-8

**Published:** 2019-01-22

**Authors:** Denis Rainko, Zoran Ikonic, Anas Elbaz, Nils von den Driesch, Daniela Stange, Etienne Herth, Philippe Boucaud, Moustafa El Kurdi, Detlev Grützmacher, Dan Buca

**Affiliations:** 1 Peter Grünberg Institute (PGI 9) and JARA-Fundamentals of Future Information Technologies, Forschungszentrum Juelich, Juelich, 52425 Germany; 2 Pollard Institute, School of Electronic and Electrical Engineering, University of Leeds, Leeds, UK; 3 Center for Nanoscience and Nanotechnologies, C2N UMR 9001 CNRS-Université Paris-Sud, Universite Paris-Saclay, Bâtiment 220, Rue Andre Ampère, F-91405 Orsay, France

## Abstract

In recent years much effort has been made to increase the Sn content in GeSn alloys in order to increase direct bandgap charge carrier recombination and, therefore, to reach room temperature lasing. While being successful for the former, the increase of Sn content is detrimental, leading to increased defect concentrations and a lower thermal budget regarding processing. In this work we demonstrate strong photoluminescence enhancement in low Sn content Ge_0.94_Sn_0.06_ layers by implementing tensile strain. Fitting of the calculated photoluminescence spectra to reproduce our experimental results indicates a strain of ~1.45%, induced via an SiN_x_ stressor layer, which is strong enough to transform the investigated layer into a direct bandgap semiconductor. Moreover, theoretical calculations, using the 8-band k·p model, show the advantages of using low Sn content tensile strained GeSn layers in respect to gain and lasing temperature. We show that low Sn content GeSn alloys have a strong potential to enable efficient room temperature lasers on electronic-photonic integrated circuits.

## Introduction

Si photonics is entering the market for short haul interconnects to construct energy efficient data centers and high performance computers. It offers high bandwidths and thus high speed data communication. In this regard, the conventional chip design is replaced or extended by optical devices like waveguides, modulators, detectors and lasers, in which information is processed via photons. For the latter, GeSn lasers have emerged as a viable solution for Si-compatible integrated group-IV laser sources^1,2^.

Ge is an indirect semiconductor with its direct conduction band valley (Γ-valley) only 150 meV above the indirect valley (L-valley). On the other hand, Sn is a semimetal with a negative bandgap at the Γ-point. By incorporating Sn into the Ge lattice both Γ- and L-valley bandgaps decrease. This effect is more pronounced for the Γ-bandgap, causing a transition (*ΔE*_*L-Γ*_ = *E*_*L*_ *– E*_*Γ*_ > *0 eV*) into a fundamental direct bandgap semiconductor at Sn contents around 8 at.% for cubic (unstrained) GeSn^3^. Introducing tensile strain into Ge has a similar effect on bandgaps as Sn incorporation^4^. Compressive strain on the other hand increases the requirements for Sn content in order to reach direct bandgap GeSn.

Although introduced in the early 80 s’, the epitaxy of GeSn alloys has recently strongly advanced, due to the introduction of new precursors into chemical vapor deposition (CVD) processes^5,6^. The ability to incorporate high Sn contents led to the proof of lasing from CVD grown bulk GeSn layers^3^. By increasing crystalline quality and Sn content, the maximum lasing temperature has continuously increased, presently reaching 180 K^7,8^. Recently, it was shown that ternary SiGeSn semiconductors are suitable barrier materials for GeSn/SiGeSn multi quantum wells (MQWs), leading to significantly improved thresholds of GeSn lasers^9,10^.

Based on 8-band k∙p calculations, it has been shown that, in the case of CVD grown high Sn content GeSn/SiGeSn heterostructures, efficient carrier confinement is limited by the low Si incorporation in the barrier^11^.

Most of present activity on GeSn alloys and devices is related to direct epitaxial growth on Si or Ge substrates^12–14^. Due to the smaller lattice constant of the Ge substrate, compared to GeSn, the epitaxial layers are under biaxial compressive strain. The strain value depends on the alloy stoichiometry and the degree of lattice relaxation of the layer. Due to the opposing effects of Sn content and compressive strain on the energy difference between Γ- and L-valley, the research target was to increase the Sn content while keeping the compressive strain low. Less attention has been paid so far to the possibilities of combining low Sn content alloys with tensile strain in order to reach direct bandgap GeSn^15,16^. In the case of Ge, gain and radiative recombination under tensile strain were studied in depth, both theoretically and experimentally^4,17,18^. For GeSn, first experiments show that tensile strain can be implemented using SiN_*x*_ stressors (commonly used in CMOS processing) to enhance photoemission for Sn contents around 8 at.%^16,19^.

In this work, the experimental results of photoluminescence (PL) analysis of GeSn microdisks under tensile strain are discussed. We show that using low Sn content layers, that initially exhibit an indirect bandgap, can be changed into efficient light sources by appropriate tensile strain engineering. The application of 1.45% tensile strain to GeSn microdisks with Sn contents as low as 6.3 at.% causes a transition into a direct bandgap alloy, inducing intensity enhancement of the PL emission by an order of magnitude. In the second part optical gain from low Sn content GeSn is investigated, discussing advantages from an epitaxial point of view, and computationally by using the 8-band k∙p model. It shows that a substantial gain is achievable at reasonably low values of applied tensile strain, even if free carrier absorption (FCA) is accounted for, and that this gain exceeds the gain achievable for commonly used high Sn content GeSn with residual compressive strain.

## Experimental

The influence of tensile strain on the optical properties was studied by growing a Ge_0.94_Sn_0.06_ layer on top of a Ge virtual substrate (Ge-VS)^20^ via reactive gas source epitaxy in an AIXTRON TRICENT reactor. The herein used precursor for Ge was digermane (Ge_2_H_6_) and tin tetrachloride (SnCl_4_) for Sn. The 265 nm thick GeSn layer was grown at 375 °C and exhibits a biaxial compressive strain of −0.32%. Sn content, thickness and strain were determined by Rutherford Backscattering Spectrometry (RBS) and X-Ray Diffraction (XRD).

**Figure 1:**
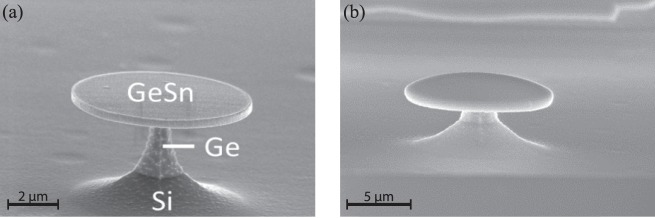
SEM images of a GeSn layer after patterning into a microdisk on a Ge pedestal (**a**), and an example of a structure after deposition of an SiN_*x*_ stressor layer (**b**).

Tensile strain is applied to the layer by using a stressor layer method as developed in a previous work where it was applied to pure Ge cavities^21,22^. The method is based on transferring strain from an SiN_*x*_ stressor layer on a microdisk surface. Starting from a 200 nm Ge_0.94_Sn_0.06_/Ge-VS layer, microdisk cavities were fabricated using standard e-beam lithography followed by two specifically developed etching processes: i) Cl_2_ based Inductively Coupled Plasma (ICP) etching used to transfer the electron beam resist pattern to the GeSn layer; ii) selectively etching of the Ge-VS using an SF_6_ gas ICP procedure. As shown in Fig. 1, freestanding GeSn layers on a Ge pedestal are obtained. At this stage, the −0.32% residual strain in the GeSn layer is fully relaxed, since it is mechanically almost decoupled from the substrate. Finally, an SiN_*x*_ stressor layer, exhibiting built-in compressive strain, is deposited by plasma-enhanced CVD (PECVD) on the top surface of the under-etched GeSn microdisks. The compressively built-in strain of the SiN_*x*_ layer transfers into tensile strain in the GeSn layer (as shown in Fig. 1b).

PL measurements are performed using a µ-PL setup, where the excitation from a continuous wave (CW) Nd:YAG laser (1064 nm wavelength) is focused into a 5–7 µm spot diameter on the sample surface by a x40 objective with numerical aperture (NA) of 0.6 and a working distance of 4.5 mm. The sample is bonded on the cold finger of a cryostat to allow cooling to cryogenic temperature. Emission from the disk is collected by the same objective from the top surface of the sample and analyzed with a monochromator spectrometer using a grating with 600 lines/mm, or a Fourier Transform Infrared (FTIR) spectrometer. In both cases, the emission is detected by an extended thermoelectric cooled InGaAs photodiode with a cutoff energy of 0.51 eV when cooled, or 0.48 eV at room temperature. The photocurrent response of the photodiode is amplified by a transimpedance amplifier analog circuit with the gain set to 10^7^ V/A. To extend the detection wavelength, the spectrum is analyzed with an LN_2_ cooled InSb detector at 80 K.

## Results and Discussion

### PL measurements

**Figure 2:**
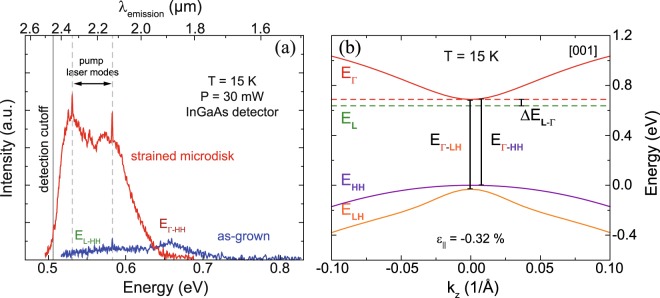
(**a**) PL spectra of the as-grown Ge_0.937_Sn_0.063_ layer (blue) and a strained 6 µm diameter microdisk (red). The sharp peaks indicated by black arrows stem from the pump laser beam. (**b**) Band dispersion of the as-grown alloy in the [001] direction at 15 K. The lowest band edge of the indirect L-valley is indicated by dashed green lines.

The PL spectrum at 15 K as obtained from the as-grown layer is shown in Fig. 2a (blue spectrum). In this case, since the GeSn layer is epitaxially bound to the substrate, the 265 nm thick partially relaxed layer has a residual compressive strain of −0.32%. Calculations reveal an indirect semiconductor, with the fundamental bandgap given by *E*_*L-HH*_ = 0.64 eV. The direct bandgap is found at *E*_*Γ-HH*_ = 0.69 eV. Consequently, the energy splitting between indirect and direct bandgap for this indirect bandgap semiconductor is *ΔE*_*L-*__*Γ*_ = −40 meV. The calculated band dispersion in the [001] direction of the as-grown layer is shown in Fig. 2b. The measured PL spectrum shows both signatures from direct bandgap transitions at 0.66 eV and indirect bandgap transitions associated to the broad contribution above 0.62 eV. This is in good agreement with the calculations discussed above.

The emission signal from the as-grown GeSn layer is weak and requires a pump power of 30 mW to detect clear PL signals with a reasonable signal-to-noise ratio. Therefore, a high sensitivity extended InGaAs detector is used. Nevertheless, this layer was used to process microdisks by underetching circular mesa structures (Fig. 1a) and depositing a SiN_*x*_ stressor layer on top (Fig. 1b), inducing tensile strain to the freestanding part of the disk. The PL of such a disk with a diameter of 6 µm was analyzed under the same experimental conditions. A strong redshift of the emission and at least one order of magnitude signal intensity enhancement (red spectrum in Fig. 2a) is detected.

**Figure 3:**
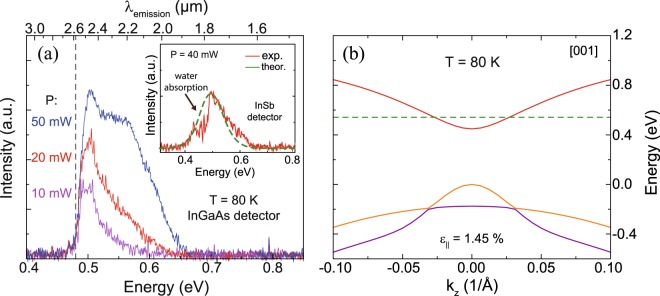
(**a**) PL spectra of the 6 µm diameter disk measured under different excitation power. The inset shows the measurement at 40 mW performed with an LN_2_ cooled InSb detector. (**b**) Band dispersion of the micro disk under 1.45% tensile strain at 80 K.

The power dependent emission spectra of the tensile strained disks, measured at 80 K with an uncooled InGaAs detector, are shown in Fig. 3a. At lower excitation power maxima close to the detection cut-off at 0.48 eV are visible. Below this region strong water absorption lines occur at the PL maxima region (inset of Fig. 3a), as measured with an InSb detector, having a cutoff energy of 0.26 eV. The PL signal is relatively broad, with an onset energy of 0.42 eV, which is assumed to correspond to the fundamental *E*_*Γ-LH*_ band gap (red spectrum in inset of Fig. 3a). Theoretical PL calculations (see method section) were performed to extract the strain in the microdisk. Assuming a tensile strain of 1.45%, the fundamental transition *E*_*Γ-LH*_ is expected at 0.411 eV. Band filling for an injection carrier density of 5 × 10^17^ cm^−3^ and an Sn content of 6.3 at.% shifts the maximum of the PL intensity to 0.49 eV (green curve in inset of Fig. 3a). Here, a homogeneous broadening with a FWHM of 25 meV was assumed (details in method section), that takes into account strain fluctuations. The resulting band dispersion in the [001] direction is shown in Fig. 3b. This is in reasonable agreement with PL results. The microdisk has a directness *ΔE*_*L-*__*Γ*_ of 94 meV, so that indirect bandgap transitions (*E*_*L-LH*_ = 0.55 eV) are negligible and do not contribute significantly to the measured PL spectrum. The same argument holds true for *E*_*Γ-HH*_ transitions, where calculations indicate a ten orders of magnitude lower intensity compared to *E*_*Γ-*LH_ transitions due to the strong splitting of HH and LH (*E*_*LH*_*-E*_*HH*_ = 184 meV). Another significant feature is the strong increase of PL in the region around 0.55 eV inducing a broadening of the spectrum to higher energies, as observed for strained disks under CW pumping (Fig. 3a). This feature can be explained by *E*_*Γ-LH*_ transitions originating from low tensile strain regions around the Ge pillar^15,16,23^ corresponding to higher bandgaps. Besides that, due to the deposition of SiN_x_ on top of the layer, a bending of the microdisk becomes visible (cf. Fig. 1b) and causes strain inhomogeneities, which in turn also could lead to a significant broadening of the spectrum. This effect can be suppressed by deposition of SiN_x_ around the whole microdisk. Band filling effects should also occur, but play a minor role in contributing to the higher energy region of the PL spectrum^24^. Therefore, the experimental comparison to compressive as-grown GeSn layers shows (i) the appearance of *E*_*Γ-*LH_ transitions with; (ii) a red shift of the PL spectrum; (iii) and a strong enhancement of the optical performance originating from the tensile strain induced by the SiN_x_ stressor. The introduction of tensile strain into the GeSn lattice clearly shows a transition from an indirect into a direct bandgap semiconductor, perceivable as a strong PL emission enhancement.

The results above motivated us to theoretically investigate lasing from low Sn content tensile strained GeSn in contrast to the present studied compressively strained high Sn content GeSn laser structures.

### Band structure calculations

**Figure 4:**
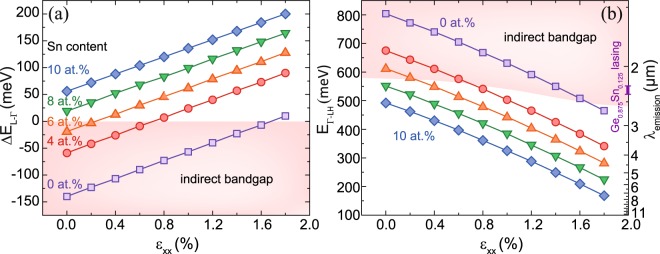
Directness *ΔE*_*L-Γ*_ (**a**), direct bandgap *E*_*Γ-LH*_ and the corresponding emission wavelengths (**b**) as they depend on tensile strain for several Sn contents at 300 K. For comparison, the lasing region of bulk Ge_0.875_Sn_0.125_ from ref.^3^ is marked on the wavelength scale.

In order to address the feasibility for light emission applications and to compare the properties of the tensile and compressively strained GeSn alloys, band structure and gain calculations were performed. A description of the theoretical framework can be found in the Method section. First, biaxial tensile strain values required to induce a direct bandgap transition for different Sn contents were determined (Fig. 4a). As expected, the required tensile strain for the transition from an indirect to a direct bandgap semiconductor decreases with increasing Sn content, approaching zero (cubic lattice) for ~7 at.% Sn. For example, for an Sn concentration *x*_*Sn*_ of 4 at.% a tensile strain of 0.70% is necessary, compared to only 0.24% for an Sn content of 6 at.%. For the later alloy, increasing the strain up to 1.70% assures a directness *ΔE*_*L-Γ*_ > 100 meV, which makes a wide range of emission wavelengths accessible, as shown in Fig. 4b. The strain value range described above lies within the experimentally accessible range using standard CMOS SiN_*x*_ stressor technology^18,25^. For Ge, our calculations predict the transition into a direct bandgap at 1.75% which is in excellent agreement with other theoretical results^17,26,27^.

For cubic GeSn – i.e. unstrained GeSn – the two highest valence bands are degenerate at the Γ-point, differing in their out-of-plane (here called z-direction) effective masses ${m}_{z}^{\ast }$ and in-plane (here called x,y-directions) effective masses ${m}_{x,y}^{\ast }$, the values of which are consistent with their names - light hole (LH) and heavy hole (HH) valence bands. ${m}_{z}^{\ast }$ and ${m}_{x,y}^{\ast }$ are equal in this case for each band respectively. When strain is applied to the lattice the degeneracy is lifted. In case of tensile strain, the LH band becomes the highest (top) valence band, while for compressive strain it will be the HH band. To be precise, the mass-based classification into LH and HH bands is correct only for the [001] direction (perpendicular to the wafer (001) surface), and can also be used for the unstrained case. In the in-plane, [100] and [010], directions, the LH effective mass ${m}_{LH,x,y}^{\ast }$ is actually larger than the HH effective mass ${m}_{HH,x,y}^{\ast }$ for strained GeSn.

**Figure 5:**
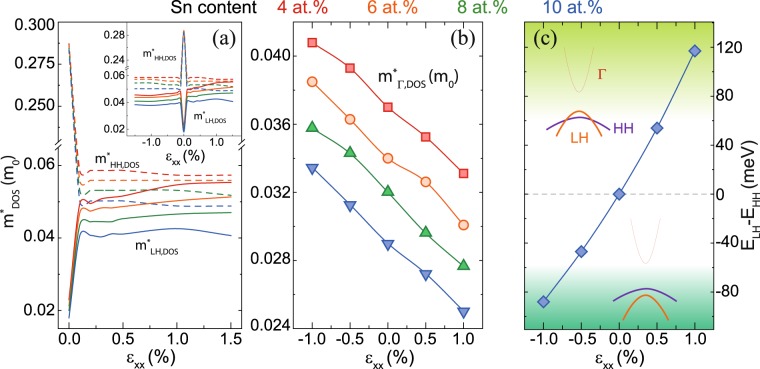
The strain-dependent HH, LH (**a**) and Γ−electron (**b**) density of states (DOS) effective masses. The inset shows the hole effective masses for an extended range of strain. (**c**) Strain dependence of valence band splitting *ΔE*_*LH-HH*_.

To discuss the advantages of tensile strain for lasing in GeSn, the net material gain was chosen as the most important figure of merit. Although gain has been calculated using the energy dispersion *E(k)* derived from 8-band k·p calculations, the DOS model with in-plane and out-of-plane effective masses is useful to discuss the influence of strain. The DOS increases with an increasing DOS effective mass ${m}_{DOS}^{\ast }$, which can be calculated using HH or LH masses as ${({m}_{x,y}^{\ast }{m}_{x,y}^{\ast }{m}_{z}^{\ast })}^{1/3}$. The in-plane effective mass clearly has a stronger influence on ${m}_{DOS}^{\ast }$. Applying tensile strain to GeSn increases the LH effective mass (Fig. 5a), while HH and Γ-electron effective masses remain constant or decrease (Fig. 5a,b). For biaxial tensile strain of 1.5% there is no significant difference between the ${m}_{HH,DOS}^{\ast }$ and ${m}_{LH,DOS}^{\ast }$, i.e. both types are equally able to provide gain (although for different polarizations of light). We should also note that the squared optical transition matrix element is somewhat larger for Γ-LH transitions for z-polarized light than for Γ-HH transitions for x- or y-polarized light (by a factor of 4/3 at the zone center), which originates from their nature in the 8-band k·p model. Except for different DOS effective masses, the valence band splitting of HH and LH bands strongly affects the gain. It is found that this splitting is independent of the Sn content. Applying tensile strain shifts the LH band above the HH band, inducing a significant valence band splitting for strain values above 0.5% (Fig. 5c).

### Material gain

**Figure 6:**
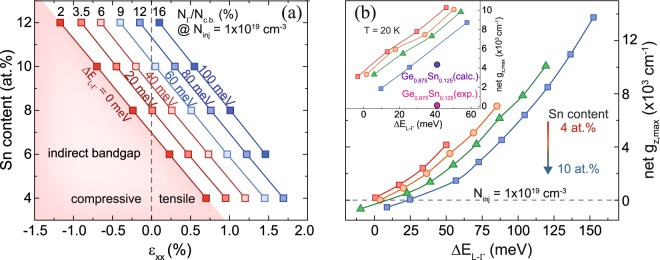
(**a**) Achievable directness *ΔE*_*L-Γ*_ depending on strain and Sn content. (**b**) out-of-plane net gain maximum *g*_*z,max*_ for several Sn contents, as it depends on the directness, at an injection carrier density of 1 × 10^19^ cm^−3^ at 300 K. Highest values of *g*_*z,max*_ are achieved for strain values of 1.5%.

Based on the above considerations, it is expected that tensile strain in low Sn content GeSn will, at least, lead to similar material gain as in compressively strained high Sn content GeSn layers, where the majority of injected holes occupy the HH band. For a reasonable comparison of these two cases, peak values of the net material gain were compared for several Sn contents, while keeping a constant directness *ΔE*_*L-Γ*_. This implies that the gain is mainly influenced by the different valence band alignments, while keeping similar conditions for the conduction band. Since *ΔE*_*L-Γ*_ depends on both, strain and Sn content, the map shown in Fig. 6a indicates the range of strain values and material compositions required for different constant values of *ΔE*_*L-Γ*_. Moreover, for each *ΔE*_*L-Γ*_ the electron population of the Γ-valley is written for a total injection carrier density *N*_*inj*_ of 1 × 10^19^ cm^−3^ and temperature of 300 K, which is a reasonable charge carrier density in the lasing regime^4,28,29^. Due to the small effective mass of the Γ-valley, compared to the L-valley, a significant directness is needed to achieve a sufficient Γ population.

Since the largest gain in tensile strained GeSn is achieved for z-polarized light, the achievable net gain *g*_*z,max*_ was investigated for a maximum tensile strain of 1.5% (Fig. 6b). When comparing gain for different Sn contents, but at a constant directness (e.g. *ΔE*_*L-Γ*_* = *50 meV), it is evident that the net gain is largest for low Sn content tensile strained alloys. For comparison, gain at 20 K is compared to theoretical and experimental values for compressively strained Ge_0.875_Sn_0.125_ material, used previously in ref.^3^ for laser demonstration (purple/pink circles in inset of Fig. 4b). The gain values calculated for low Sn content GeSn are considerably higher than for Ge_0.875_Sn_0.125_. It should be noted that the theoretical and experimental values of gain in Ge_0.875_Sn_0.125_ differ significantly, which may be due to neglecting Auger recombination in calculations, as well as the influence of defects in high Sn content GeSn. In this respect we are inclined to believe that, for a constant directness, lower Sn content tensile strained alloys are preferable for achieving high gain values, because the crystalline defect density is strongly reduced in low Sn content alloys. A strain of e.g. 1.5% in Ge_0.94_Sn_0.06_ – which is comparable to the experimentally achieved strain – guarantees a high directness at a reasonable conduction band population of electrons and offers gain values of above 7000 cm^−1^. Extending these results to even lower or no Sn content, the highest gain value for a constant directness can be achieved for Ge, but a directness of 50 meV translates into a biaxial strain of 2.3%, which was experimentally never demonstrated for Ge and would be very difficult to achieve.

### Lasing temperature and injection current thresholds

**Figure 7:**
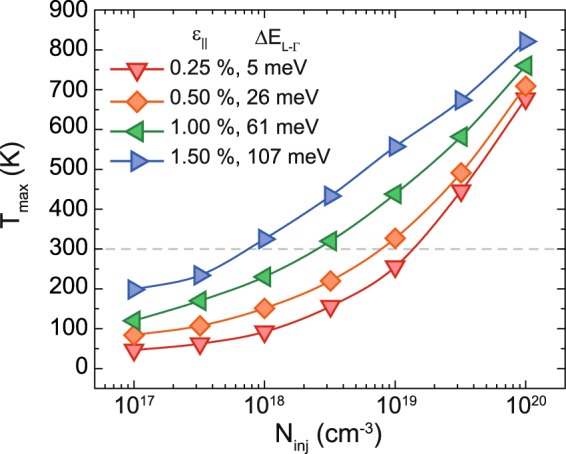
Injection carrier density dependent temperature thresholds *T*_*max*_ of Ge_0.94_Sn_0.06_ for different strain values and the corresponding directnesses.

In the last few years, the maximum lasing temperature *T*_*max*_ of GeSn alloys – at which the gain becomes zero – constantly increased with Sn content and, hence, T_max_ increased with the directness^3,7,8,30^. In our approach the directness is increased by introducing tensile strain. The impact on *T*_*max*_ can be seen in Fig. 7. We calculated *T*_*max*_ for different injection carrier densities *N*_*inj*_ and strain values (0.25% ≤ ε_||_ ≤ 1.50%) at a fixed Sn content of 6 at.%. Keeping in mind the goal of room temperature lasing (dashed line), tensile strain enables room temperature lasing at low injection carrier densities. At a tensile strain of e.g. 1.50% an *N*_*inj*_ of ~1 × 10^18^ cm^−3^ is needed to achieve lasing at 300 K. Increasing *N*_*inj*_ increases gain losses due to FCA but at the same time the material gain is increased drastically, which shifts *T*_*max*_ to higher values. Since other possible loss mechanisms, such as Auger recombination, are not taken into account in these calculations, the experimentally required values will be most likely shifted to higher injection carrier densities than predicted here.

In addition to the above results, we have calculated threshold current densities using the structure presented in ref.^31^, where the threshold injection carrier density *N*_*inj,th*_ was determined at net material gain values around 0 cm^−1^. The lifetimes *τ*_*rad*_, *τ*_*SRH*_ and *τ*_*Aug*_ chosen for radiative, Shockley-Read-Hall and Auger processes are discussed in the Method section.

**Figure 8:**
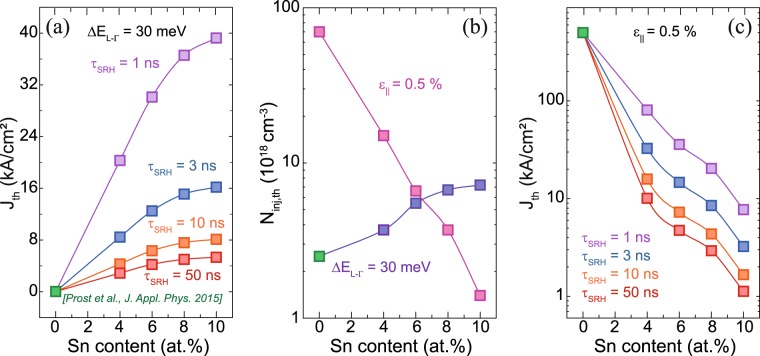
Sn content dependent threshold current densities for (**a**) a constant directness of 30 meV and (**c**) a constant biaxial strain of 0.5% for different Shockley-Read-Hall lifetimes τ_SRH_. (**b**) Corresponding threshold carrier densities for (**a**) and (**c**). As a reference point for Ge data from ref.^31^ was taken.

In order to be able to compare the results for different Sn contents, at first the directness was fixed to 30 meV. In this case, the threshold current density increases with Sn content reaching values up to 16 kA/cm² for a *τ*_*SRH*_ of 3 ns, as shown in Fig. 8a. Increasing *τ*_*SRH*_ leads to values similar to reported values for Ge in ref.^31^ (0.015 kA/cm² at *ε*_*||*_* = *2%). The corresponding carrier density thresholds are independent of *τ*_*Aug*_ and *τ*_*SRH*_ and are found to be in the range of 1 × 10^18^ cm^−3^ as shown in Fig. 7b. The explanation for this behavior is the change in the HH/LH splitting *ΔE*_*LH-HH*_. Increasing the Sn content at a fixed directness is only achievable when the strain changes from tensile (corresponding to low Sn contents) to compressive (corresponding to high Sn contents). This increases the impact of HH states on gain, which now becomes more evenly distributed between x- and z-polarizations, and therefore a higher pumping is required to achieve gain. This also implies that for keeping the valence band splitting of HH and LH constant and increasing the Sn content, the directness will predominantly be increased and plays the major role affecting gain. A decreasing *J*_*th*_ can be expected as shown in Fig. 8c for a constant strain of 0.5%. In this context, at an Sn content of 6 at.%, *J*_*th*_ below 10 kA/cm² can be achieved to maintain positive net gain.

## Conclusion

This work presents the possibilities of implementing tensile strain into low Sn content GeSn. Experimentally, we could show, by applying tensile strain using SiN_*x*_ stressor layers, a strong increase of PL as a consequence of an indirect to direct bandgap transition. Fitting PL measurements at 80 K we extract a tensile strain of ~1.45% corresponding to a directness of 94 meV for GeSn alloys with 6 at.% Sn. Band structure and gain calculations using the 8-band k·p method were employed to investigate the influence of tensile strain on gain. We conclude that, at a given constant directness, lower Sn content alloys under tensile strain offer higher gain values compared to in literature studied compressively strained high Sn content alloys. This is related to the strain-dependent change of the valence band configuration. Furthermore, low Sn content GeSn is predicted to be a promising material to achieve lasing above room temperature for injection carrier densities exceeding 1 × 10^18^ cm^−3^. Considering the fact that low Sn content GeSn is epitaxially and in terms of CMOS processing more favorable and possesses lower crystalline point defect densities in comparison to high Sn content layers, we suggest the implementation of tensile strained low Sn content GeSn into lasing architectures.

## Methods

### Band structure calculations

To investigate the influence and possible benefits of applying tensile strain to bulk GeSn layers, band structure calculations have been performed at 300 K. High Sn content (10 at.%), compressively strained GeSn layers – which are already epitaxially accessible – were compared to low Sn content tensile strained GeSn alloys with Sn contents *x*_*Sn*_ below 12 at.% and strain values ε_xx_ of −1.5% < ε_xx_ < 1.5%. Bandgaps at the relevant Γ- and L-points for unstrained material were calculated using an elemental concentration dependent interpolation of the elemental bandgaps ${E}_{i}^{{\rm{\Gamma }},L}$, with bowing parameters ${b}_{i}^{{\rm{\Gamma }},L}$:

1 $${E}^{{\rm{\Gamma }},L}={E}_{Ge}^{{\rm{\Gamma }},L}(1-{x}_{Sn})+{E}_{Sn}^{{\rm{\Gamma }},L}{x}_{Sn}-{b}_{GeSn}^{{\rm{\Gamma }},L}(1-{x}_{Sn}){x}_{Sn}.$$

Bowing parameters were derived from empirical pseudopotential method and PL measurements on bulk GeSn. All calculations have been performed assuming a (001) substrate surface. Temperature dependencies of bandgaps were implemented for all constituents via Varshni’s formula, while deformation potentials were used to account for strain effects. All the material parameters used in calculations in this work can be found in ref.^11^.

### Spontaneous emission and gain calculations

The strain in the processed microdisk with a diameter of 6 µm was derived by fitting PL measurements performed with an InSb detector cooled at 80 K at 40 mW pump power. The model used to calculate the spontaneous emission (photoluminescence spectrum due to direct and indirect recombination) was taken from ref.^17^, Eqs (8) and (12). The presence of any broadening, e.g. coming from carrier scattering, can be accounted for by finding the convolution of the calculated spectrum with the broadening function (Lorentzian in case of homogeneous broadening, with appropriate FWHM). Any inhomogeneous strain effect on both the band structure and band energies on absolute scale (therefore inducing carrier spatial redistribution) is difficult to include precisely in these calculations, but can be approximately included as another contributor to the overall “effective” homogeneous broadening, and this simple model was employed in the presented calculations. In the here presented PL calculations we used a homogeneous broadening of 25 meV.

The calculated bandgaps related to the measurements shown in Fig. 2a and in Fig. 3a are summed up in Table 1.

**Table 1:** Extracted and calculated bandgaps from PL calculations for Ge_0.94_Sn_0.06_.

x_Sn_ (at.%)	ε_||_ (%)	T (K)	ΔE_Γ-LH_ (meV)	ΔE_Γ-HH_ (meV)	ΔE_L-LH_ (meV)	N_inj_ (cm^−3^)
6.3	−0.32	15	725	694	642	—
6.3	1.45	80	411	598	505	—
6.3	1.45	80	492	676	548	5 × 10^17^

The influence of the different strain conditions on optical properties were investigated by material gain calculations using the 8-band k·p model and equation 9.1.27 from ref.^32^ without taking into account broadening:

2 $$g(\hslash \omega )=\frac{\pi \omega }{{n}_{r}c{\varepsilon }_{0}}\frac{2}{V}\sum _{{k}_{v}}\sum _{{k}_{c}}{|\hat{e}\cdot {{\boldsymbol{\mu }}}_{cv}|}^{2}\delta ({E}_{c}-{E}_{v}-\hslash \omega )({f}_{{\rm{c}}}(k)-{f}_{v}(k)),$$

with *n*_*r*_ being the refractive index, *ω* the radiation frequency and ${|\hat{e}\cdot {{\boldsymbol{\mu }}}_{cv}|}^{2}$ the interband momentum matrix element. *f*_*c*,*v*_ are the Fermi-Dirac distributions for electrons in the conduction and valence band, with the quasi-Fermi levels *F*_*c*,*v*_, and *E*_*c*_ and *E*_*v*_ come from the 8-band k·p Hamiltonian. Same as for the spontaneous emission, a simple model to include the effects of any additional broadening would be to find the convolution of this spectral profile with appropriate Lorentzian.

The calculated material gain was corrected for free carrier absorption (FCA), which was calculated using the second order perturbation model described in ref.^33^. In this model acoustic phonon scattering, deformation potential scattering (L-valley), intervalley scattering, ionized impurity scattering and alloy scattering are included.

### Threshold current densities

Net material gain calculations in this work explicitly include only the losses from free carrier absorption, so that a basic understanding of the influence of material composition on current threshold is provided. Therefore, losses from Shockley-Read-Hall, Auger recombination and cavity losses were neglected. The radiative lifetime *τ*_*rad*_ is in the range of 20–50 ns and was derived from PL calculations, where the total spontaneous radiative recombination rate, including both direct and phonon-assisted indirect transitions, was calculated as in ref.^17^. The Auger lifetime *τ*_*Aug*_ was taken from ref.^31^ for Ge and kept constant at 10 ns, while the Shockley-Read-Hall lifetime *τ*_*SRH*_ was varied between 1 ns (3 ns in ref.^31^ for Ge) and 50 ns. The latter is unknown for GeSn but is expected to be lower than in Ge because of larger defects density. This gives a total carrier lifetime of $\tau ={(1/{\tau }_{rad}+1/{\tau }_{Aug}+1/{\tau }_{SRh})}^{-1}$.
